# Comprehensive Study on Chemical and Hot Press-Treated Silver Nanowires for Efficient Polymer Solar Cell Application

**DOI:** 10.3390/polym9110635

**Published:** 2017-11-22

**Authors:** Yang-Yen Yu, Yo-Jen Ting, Chung-Lin Chung, Tzung-Wei Tsai, Chih-Ping Chen

**Affiliations:** 1 Department of Materials Engineering, Ming Chi University of Technology, No.84, Gongzhuan Rd., Taishan Dist., New Taipei 24301, Taiwan; a1811227@gmail.com (Y.-J.T.); h56h913440@gmail.com(C.-L.C.); moto123robert886@gmail.com (T.-W.T.); 2 Department of Chemical and Materials Engineering, Chang Gung University, No. 259, Wenhua 1st Rd., Guishan Dist., Taoyuan 33302, Taiwan

**Keywords:** bolsilver nanowire, organic photovoltaic, transparent electrode

## Abstract

In this study, chemical treatment (CT; oxidation–reduction method) and physical treatment (HP; hot-pressing methods) were applied to improve the performance of silver nanowire (AgNW)-derived electrodes on a glass or flexible polyethylene terephthalate (PET) substrate. The four-point probe method, UV-Vis spectroscopy and scanning electron microscopy (SEM) were used to measure the properties of AgNW electrodes and compare them with those of indium tin oxide (ITO) electrodes for exploring the possibility of using CT- and HP-based AgNW electrodes for polymer solar cell (PSC) applications. Using the CT or HP method, the sheet resistance of electrodes decreased to lower than 40 Ω sq^−1^ with an average high transmittance of more than 80%. Furthermore, HP reduced the surface roughness of AgNWs, which solved the inter-electrode short circuiting problem for devices. We studied the performance of poly(3,4-ethylenedioxythiophene)-poly(styrene sulfonate) and zinc oxide-based PSC devices. The power conversion efficiency of HP-AgNW-derived poly[4,8-bis(5-(2-ethylhexyl)thiophen-2-yl)benzo[1,2-b;4,5-b′]dithiophene-2,6-diyl-alt-(4-(2-ethylhexyl)-3-fluorothieno[3,4-b]thiophene-)-2-carboxylate-2-6-diyl] (PTB7-Th):[6,6]-phenyl-C_71_-butyric acid methyl ester (PC_71_BM) devices was 7.83%, which was slightly lower than the performance of the device using ITO (8.03%) as a substrate. After a bend test (100 times) at a 2-cm curvature radius, the efficiency of AgNW/PET-derived PSCs was more than 70%. The performance of PSCs made with AgNWs and ITO electrodes is comparable, but the cost of using AgNWs for electrodes is much lower; therefore, HP-derived AgNWs demonstrate great potential for optoelectronic applications.

## 1. Introduction

Most studies on organic polymer solar cells (PSCs) have primarily focused on large-area processes at low costs and temperatures and explored electrodes that are more conductive than indium tin oxide (ITO) [1,2,3]. ITO is a transparent conductive oxide (TCO) commonly used as a transparent electrode in various optoelectronic devices; however, ITO electrodes require high vacuum processes and high temperatures (>300 °C), resulting in high production costs and also severely limiting the choice of substrates. On glass substrates, ITO sheet resistance can reach 15 Ω sq^−1^. However, this brittle characteristic limits the application of ITO on polyethylene terephthalate (PET) or polyethylene-naphthalate substrates. Other TCOs, such as SnO_2_ fluoride and zinc oxide (ZnO)-mixed aluminum, have similar issues when used on plastic substrates [4]. Carbon-based materials, such as graphene and carbon nanotubes, are considered to have high potential in replacing TCO for the application in PSCs because of their unique properties. However, the sheet resistance and optical transparency of these carbon-based electrodes are still inferior to those of ITO. A low-dimensional metal, two-dimensional ultrathin film and one-dimensional metal nanowire (NW) are also considered to be suitable electrode materials because of their favorable optical properties and stability. Peumans and Triambulo et al. investigated treated silver (Ag) NW electrodes as a promising alternative to ITO material [5,6]. Since then, various metal NWs (e.g., copper and AgNW) have been extensively studied, and several crucial variables, such as wire size, stable dispersion, wire geometric shape and aspect ratio, have been reported to exert a considerable effect on electrode performance. In addition, the small molecules of dispersive agents (such as organic polymers) may increase contact resistance between two NWs, thus increasing the optical properties of NW electrodes [7,8,9,10,11]. To reduce cross-resistance between NWs, numerous methods have been studied. Lu et al. used an acid precursor with silver ions to form a silver atom through a redox reaction in an NW structure (selective growth) and significantly reduced sheet resistance (*R*_s_). Through the selective growth of Ag, *R*_s_ was significantly reduced [12]. The use of electrically-conductive adhesives, such as poly(3,4-ethylenedioxythiophene)-poly(styrene sulfonate) (PEDOT:PSS) and ZnO, is another method to enhance the efficiency of NW electrodes. This is because adhesives not only improve bonding between wires and thus contact resistance [13,14], but also simultaneously improve the adhesion of NW electrodes to a substrate. [15] Hot-pressing (HP) (or cold pressing) is another method to effectively reduce the sheet resistance and roughness of AgNW electrodes. Khaligh and Goldthorpe used a roller hot press machine to prepare PET/AgNW electrodes at a low temperature of 80 °C. This low temperature process successfully reduced the roughness of Ag wires and did not affect sheet resistance and transparency [16]. Tokuno et al. used a high pressure and low temperature process to reduce the roughness of an AgNW electrode. The experimental results showed that the AgNW network was successfully pressed to a thickness of one wire diameter to achieve a sheet resistance of 8.6 Ω sq^−1^, and the performance of the solar cell was higher than that on an ITO electrode [17].

Many studies on AgNW-derived electrodes have been conducted [18,19]. Jiu et al. used conductive PEDOT:PSS to cover their NW network, whereas Choi et al. used sprayed AgNWs and PEDOT:PSS to prepare their AgNW electrodes. Their results showed that the transparency of the prepared electrodes was only slightly decreased. After the electrodes were heated, AgNWs were softened to reduce their roughness. The sheet resistance was reduced to 5 Ω sq^−1^, even if the resistance did not increase significantly under a bend test, showing excellent performance [20]. However, the acidity and uneven structure of PEDOT:PSS caused various degradation mechanisms and limited the device’s life [21,22,23,24]. In addition, the electrical properties of AgNW electrodes deteriorated after prolonged exposure to PEDOT:PSS [25]. Chen et al. transferred the transparent electrode of the graphene monolayer on AgNWs through HP to reduce contact resistance between the wires and fill the gap between the wires with conductive graphene (sheet resistance = 13 Ω sq^−1^). The composite electrode prepared using this method had a 14 Ω sq^−1^ resistance and 90% transmittance at 550 nm [26]. Deng et al. followed this idea by developing a large-scale production of a roll-to-roll process in which a graphene monolayer was hot pressed onto a PET substrate with AgNWs at 100 °C to obtain a continuous and uncracked graphene monolayer, which significantly improved the corrosion stability of the electrodes and adhesion of AgNWs to the substrate. The electrode had an excellent sheet resistance of 10 Ω sq^−1^ and 84% transmittance at 550 nm; these characteristics did not change even after undergoing 1000 bending cycles to a 20-mm radius of curvature [27]. Many studies on the application of AgNWs in solar cells have also been reported. Leem et al. investigated the effect of sprayed PEDOT:PSS thickness on AgNWs and solar energy efficiency. They found that the value of the fill factor could be considerably increased in the case of greater thicknesses [28]. Wang et al. fabricated a flexible bottom cell of AgNWs/PEDOT:PSS on a flexible PSC. The PET substrate was modified using acrylic resin and annealed at 80 °C. The NWs sank into the resin layer to reduce the electrode roughness, causing the performance to increase by 32%, and even after 60 bending cycles, no obvious change in cell performance was observed [29]. 

Single AgNWs have extremely high conductivity; however, because of contact resistance between AgNWs, the sheet resistance of an AgNW-based electrode is several orders of magnitude lower. Generally, the diameter and length of AgNWs are in the scale of dozens of nanometers (>50 nm) and from several to dozens of micrometers, respectively. AgNW-based electrodes are subjected to high levels of roughness (more than dozens of nanometers), which limits the performance, stability and reproductivity of derived optoelectronic devices. Therefore, overcoming this problem and fabricating an AgNW electrode with high transparency and conductivity have been the topics of research. We adopted both chemical treatment (CT; oxidation–reduction method) and physical treatment (HP; hot-pressing methods) to modify AgNW electrodes and evaluate the advantages and disadvantages of HP-and CT-based electrodes for PSC application.

## 2. Experimental Section

### 2.1. Materials

The following compounds were used as active layer materials: poly(3-hexylthiophene) (P3HT, 99.99%, UniRegion Bio-Tech Co., Hsinchu, Taiwan), poly[(5,6-difluoro-2,1,3-benzothiadiazol-4,7-diyl)-alt-(3,3‴-di(2-octyldodecyl)-2,2′;5′,2″;5″,2‴-quaterthiophen-5,5‴-diyl)] (PffBT4T-2OD, 99.99%, 1-Material), poly[4,8-bis(5-(2-ethylhexyl)thiophen-2-yl)benzo[1,2-b;4,5-b′]dithiophene-2,6-diyl-alt-(4-(2-ethylhexyl)-3-fluorothieno[3,4-b]thiophene-)-2-carboxylate-2-6-diyl] (PTB7-Th, [PCE10, PBDTTT-EFT], 99.99%, 1-Material), [(6,6)-phenylC_61_-butyric acid methyl ester] (PC_61_BM, 99.99%, UniRegion Bio-Tech Co., Hsinchu, Taiwan) and [6,6]-phenyl-C_71_-butyric acid methyl ester (PC_71_BM, 99.99%, UniRegion Bio-Tech Co, Hsinchu, Taiwan). Ascorbic acid (99.99%, Sigma-Aldrich, MO, USA) was used as a reducing agent. PEDOT:PSS (1.3–1.7 wt % (in water), UniRegion Bio-Tech Co., Hsinchu, Taiwan) was used as a hole transport layer. AgNWs with a diameter of 40–50 nm and an average length of 30 μm were purchased from Zhejiang Kechuang Advanced Materials. 

### 2.2. Modification and Processing of AgNWs

**Scheme 1:**
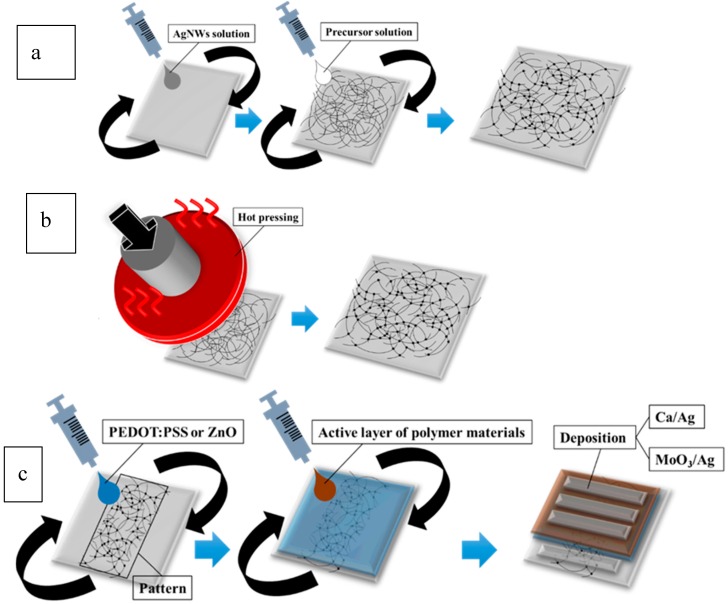
(**a**) Schematic of chemical treatment (CT); (**b**) hot-pressing (HP) treatment of AgNWs; (**c**) the fabrication of the polymer solar cell (PSC) device.

In this experiment, both CT and HP methods were used to modify AgNWs. The CT method was used to complete the AgNW node welding through a redox reaction. First, the substrate was cleaned with acetone and isopropanol (IPA). AgNWs were welded using silver nitrate (AgNO_3_), ethanol, nitric acid (HNO_3_) and ascorbic acid to form an acidic alcohol-based solution with a pH value of 3 [12]. To form a stable and conductive AgNW electrode, the acidic alcohol-based solution was dripped on the AgNW conductive film for wetting. Subsequently, the AgNW film was rotated immediately to remove the excess solution; only a small amount of solution remained in the intersection of AgNWs, which triggered the reduction of Ag ions and the deposition of Ag atoms. The Ag atoms welded the Ag wires to form a stable AgNW network electrode (Scheme 1a). Hot-pressing (HP) was used for the physical treatment. First, the AgNW dispersive solution was spin-coated on the substrate. The pressure was set to approximately 1.5 kgf/cm^2^ with various temperatures and time periods. The AgNW nodes were melted under the high temperature and high pressure, which caused the welding of AgNWs and the formation of a stable AgNW network electrode (Scheme 1b).

### 2.3. Device Fabrication

Both PSCs with the structure Ag/Ca/active layers/PEDOT:PSS/AgNW/substrates and the inverted structure Ag/MoO_3_/active layers/ZnO/AgNW/substrates were fabricated. ZnO (self-synthesized) was used as an electron transport layer, which was formed on the AgNW film through spin coating at 2000 rpm for 30 s, followed by annealing at 160 °C for 20 min. PEDOT:PSS was used as the hole transport layer, which was formed on the AgNW films through spin coating at 2000 rpm for 30 s, followed by annealing at 120 °C for 20 min. Various blend films were used as active layers for devices, including P3HT:PC_61_BM, PTB7-Th:PC_71_BM and PffBT4T-20D:PC_71_BM, which were all spin-coated at 450 rpm/30 s, followed by a second stage of spin coating at 1200 rpm/2 s for P3HT:PC_61_BM, 2000 rpm/30 s for PTB7-Th:PC_71_BM and 800 rpm/30 s for PffBT4T-20D:PC_71_BM. PffBT4T-20D: PC_71_BM received a third stage of spin coating at 1200 rpm/2 s. The annealing was performed at 140 °C for 20 min. The metal electrodes, Ca 30 nm/Ag 100 nm for normal PSCs and MoO_3_ 3 nm/Ag 100 nm for inverted PSCs, were prepared through vapor deposition (Scheme 1c).

## 3. Results and Discussion

**Figure 1:**
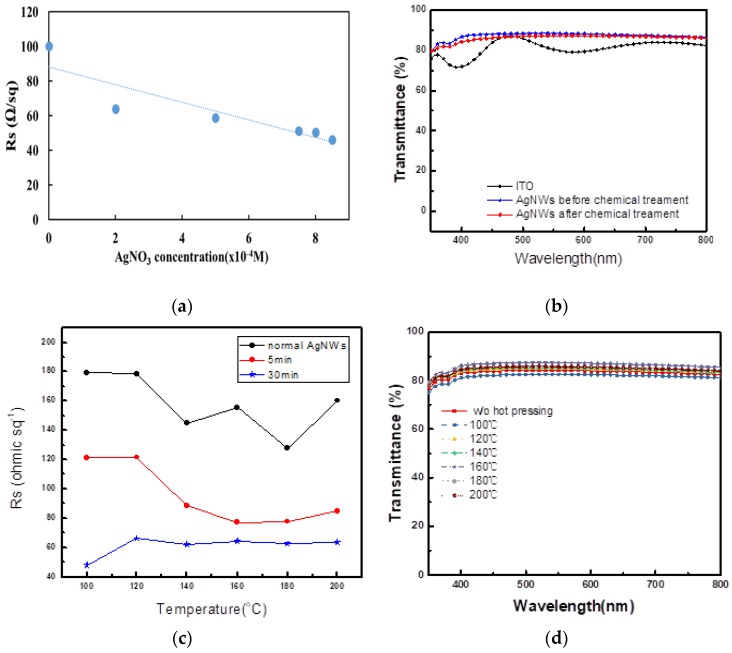
(**a**,**c**) *R*_s_ values of AgNWs: (**a**) treated at different concentrations of silver nitrate; and (**c**) applied with various temperature and time periods; and (**b**,**d**) the UV-Vis spectra of (**b**) CT-AgNWs and ITO; (**d**) HP-AgNW electrodes.

Fabricating an AgNW-based transparent electrode that simultaneously has high conductivity and transparency, as well as low surface roughness was challenging. Surface roughness and poor adhesion to the substrate resulted in unsatisfactory stability of derived optoelectronic devices. Here, AgNWs were treated separately using CT(oxidation–reduction method) and HP (hot-pressing method) to overcome these disadvantages. We fabricated the normal AgNW electrode from the IPA-diluted solution. Through optimizing the concentration and the spin-coating rates of the AgNW solution, we obtained a sheet resistance (*R*_s_) of 100–180 Ω sq^−1^ and an average transmittance of 85% (denoted as a normal AgNW electrode). Because the values of the *R*_s_ and transmittance were trade-offs, we evaluated the effects of the CT and HP treatments on the optoelectronic properties of electrodes and compared them with the normal AgNW electrode. First, we modified the normal AgNW electrode by using CT and used ascorbic acid as a reducing agent to reduce Ag ions into Ag atoms; the nitrate ions were introduced into the solution to prevent the rapid precipitation of Ag ions. The optimized CT process allowed Ag ions mainly to precipitate between the AgNW nodes and form well-defined AgNW network films. We scanned the concentration of AgNO_3_ and evaluated its effect on the *R*_s_ and transparency of the AgNW electrodes. As shown in Figure 1a, the *R*_s_ values of the AgNW films decreased significantly with an increase in the AgNO_3_ concentration. Using an optimized AgNO_3_ concentration of 8 × 10^−4^ M, we fabricated the CT-based AgNW electrode with an *R*_s_ lower than 60 Ω sq^−1^, which was 40–50% lower than that of the normal AgNW electrode. We further compared the optical transmittance of the ITO and AgNW electrodes before and after CT. We found no significant difference in optical transmittance. The transmittance in the visible region was higher than 80% (Figure 1b) before and after CT and was higher than that obtained from ITO glass. Figure 1c shows the *R*_s_ values of AgNW films treated with different HP treatments. We used a pressure of 1.5 kgf cm^−2^ and tested various process temperatures of 100 °C, 120 °C, 140 °C, 160 °C, 180 °C and 200 °C, each with time periods of 5 and 20 min. The initial *R*_s_ values of AgNWs without treatment were between 130 and 180 Ω sq^−1^. We observed a decrease in the *R*_s_ values (when compared with their initial *R*_s_ values) for 100 °C, 120 °C, 140 °C, 160 °C, 180 °C and 200 °C (with 5-min treatment) samples by 32%, 32%, 38%, 50%, 39% and 47%, respectively. The decrease in the *R*_s_ value for the electrodes treated for 30 min was 77%, 63%, 57%, 59%, 51% and 60% under the temperatures of 100 °C, 120 °C, 140 °C, 160 °C, 180 °C and 200 °C, respectively. The results suggest that the key factor in determining the *R*_s_ value of the HP process is the time period. When the HP time period was long enough, AgNWs became well welded, and thus, the contact resistance and *R*_s_ decreased under the appropriate HP energy and pressure. Figure 1d shows the UV-Vis spectra of glass/AgNW films treated at different HP temperatures. The results demonstrate that the optical transmittance of both AgNW films was not affected by the HP temperature. The optical transmittance of glass/AgNWs could reach more than 80% in the visible light region.

**Figure 2:**
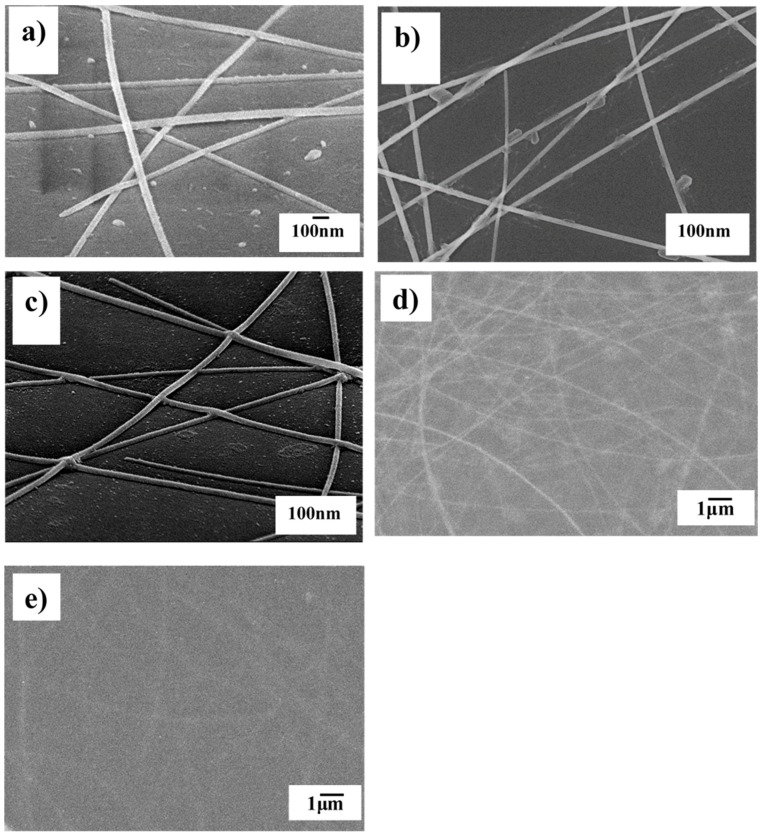
SEM images of: (**a**) glass/AgNWs; (**b**) glass/CT-AgNWs; (**c**) glass/HP-AgNWs; (**d**) glass/HP-AgNW/PEDOT:PSS/P3HT:PC_60_BM; and (**e**) glass/HP-AgNW/PEDOT:PSS/poly[(5,6-difluoro-2,1,3-benzothiadiazol-4,7-diyl)-alt-(3,3‴-di(2-octyldodecyl)-2,2′;5′,2″;5″,2‴-quaterthiophen-5,5‴-diyl)] (PffBT4T-2OD): poly[4,8-bis(5-(2-ethylhexyl)thiophen-2-yl)benzo[1,2-b;4,5-b′]dithiophene-2,6-diyl-alt-(4-(2-ethylhexyl)-3-fluorothieno[3,4-b]thiophene-)-2-carboxylate-2-6-diyl] (PTB7-Th):[6,6]-phenyl-C_71_-butyric acid methyl ester (PC_71_BM).

Figure 2a,b displays the scanning electron microscopy (SEM) images of the normal and CT-derived AgNW network. We could clearly observe the node formation at the junction of AgNWs, confirming that Ag atoms obtained from the reduction of Ag^+^ ions are precipitated in the intersection between AgNWs; thus, the CT-based AgNWs simultaneously have high conductivity and transparency. Considering the stacks of AgNWs, the estimated highest topography of the electrode is more than 150 nm (from three stacks of AgNWs) for normal and CT-AgNW electrodes. Because of the high surface roughness of these AgNW electrodes, fabricating optoelectronic devices with high reproducibility is challenging (Figure 2a,b). To further eliminate the effect of large surface roughness and reduce the *R*_s_ of AgNW films, we hypothesized that the HP process helps to form a smoother surface and maintain performance. We measured the SEM image of the HP sample treated at a temperature of 100 °C for 30 min with a pressure of 1.5 kgf/cm^2^. As shown in Figure 2c, we clearly observed the formation of nodes at the junctions between the AgNWs. Because of the efficient connection between individual AgNWs through the HP process, we obtained a decrease in *R*_s_ from 180 Ω/sq down to 50 Ω/sq (compared with normal AgNWs), which was close to the *R*_s_ value (10 Ω/sq) of the ITO glass. Compared with CT-AgNWs, the HP-AgNWs treatment showed a stronger welding effect, and the derived AgNW network was rather flatter than that for CT-AgNWs.

Figure S1 shows the taping mode atomic force microscopy (AFM) images of the AgNWs and HP-AgNWs films. The root mean square surface roughness values were 20.2 and 11.7 nm for the AgNWs and HP-AgNWs films, respectively. Smooth surface morphology was observed for the HP-AgNWs-derived film.

**Figure 3:**
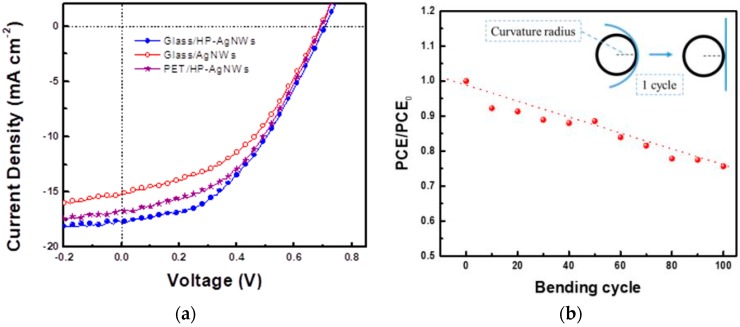
(**a**) *J*-*V* curves of PSCs based on PffBT4T-2OD:PC_71_BM using different electrodes; (**b**) bend test of the flexible PSCs (curvature radius = 2 cm; bend test value = 100 times); the inset shows the schematic of the bend test.

We first fabricated the glass/CT-AgNWs/PEDOT:PSS/P3HT:PC_61_BM (approximately 140 nm)/Ca/Ag PSC devices. Because of the high transparency and conductivity of the optimized CT-AgNWs, we observed the highest power conversion efficiency (PCE) of 2.8% (Air Mass, AM 1., 100 mW cm^−2^), which was close to that of the ITO-based device (3.5%). Nevertheless, because of the large surface roughness of CT-AgNWs, the reproductivity was low, with six of the 10 devices short circuiting. We fabricated the HP-AgNW-based (ca. 50 Ω sq^−1^) P3HT device and obtained a PCE of 2.96%, which was 16.5% higher than the normal AgNW-based (120 Ω sq^−1^) device (PCE = 2.54%), because of the increase in the fill factor (FF) value benefiting from the low *R*_s_ of the electrode (Table 1). Figure 2d shows the SEM image of glass/HP-AgNWs/PEDOT:PSS/P3HT:PC_60_BM. Because the optimized thickness of the P3HT: PC_60_BM blend film was 140 nm, we could clearly observe the fuzzy patterns of AgNWs embedding in PSC devices, and some of the AgNWs seemed prominent from the surface of the active layer. We further fabricated a thicker active layer-based (PffBT4T-2OD:PC71BM (thickness of ca. 250–300 nm)) device. As shown in Figure 2e, when we applied this thick active layer, we could barely see the image of AgNWs, which suggested a lower possibility of protruding AgNWs making contact with the upper electrode and causing a short circuit problem. Table 1 displays the PCEs of the PffBT4T-2OD-derived devices. The PCEs of AgNWs and HP-AgNW PffBT4T-2OD-derived devices were 4.21 and 5.33%, respectively (Figure 3). A higher PCE was observed for HP-AgNWs primarily because of the higher RF value attributed to the lower *R*_s_ value of the electrode.

To evaluate the HP-AgNWs for flexible PSCs, we optimized the fabrication condition of the AgNW-PET substrate. Because the glass transition temperature of PET is ca. 100 °C, we applied the temperatures of HP at 80 °C, 90 °C and 100 °C for 10 min and then for 30 min. Figure S2 displays the *R*_s_ values of electrodes before and after various HP conditions. The *R*_s_ of the electrode decreased by 40–50% under the HP process. Figure S3 shows the UV-Vis spectra of these electrodes; the transmittance is almost the same before and after HP treatment, indicating the realizability of HP-AgNW electrodes. Figure S4 shows the SEM images of PET/AgNW/PEDOT:PSS films (left side: AgNWs; right side HP-AgNWs (80 °C, 10 min)); we observed the same phenomenon as the glass substrate. We found the flattened surface of HP-AgNWs and some of the AgNWs fell onto or embedded in the surface of the PET. Table 1 displays the performance of the PET-based device. We obtained a PCE of 5.09% for the PffBT4T-2OD device, which was similar to the glass-based device (Figure 3a). Figure 3b illustrates a bend test of the flexible PSC; the schematic of the bend test is displayed. We applied a curvature radius and bend test value of 2 cm and 100-times, respectively. The bend test shows that the original efficiency of PET-based PSCs can be maintained by more than 70% after the bending cycles. This result shows that the HP-AgNW electrodes have great potential for application in flexible optoelectronics. 

**Figure 4:**
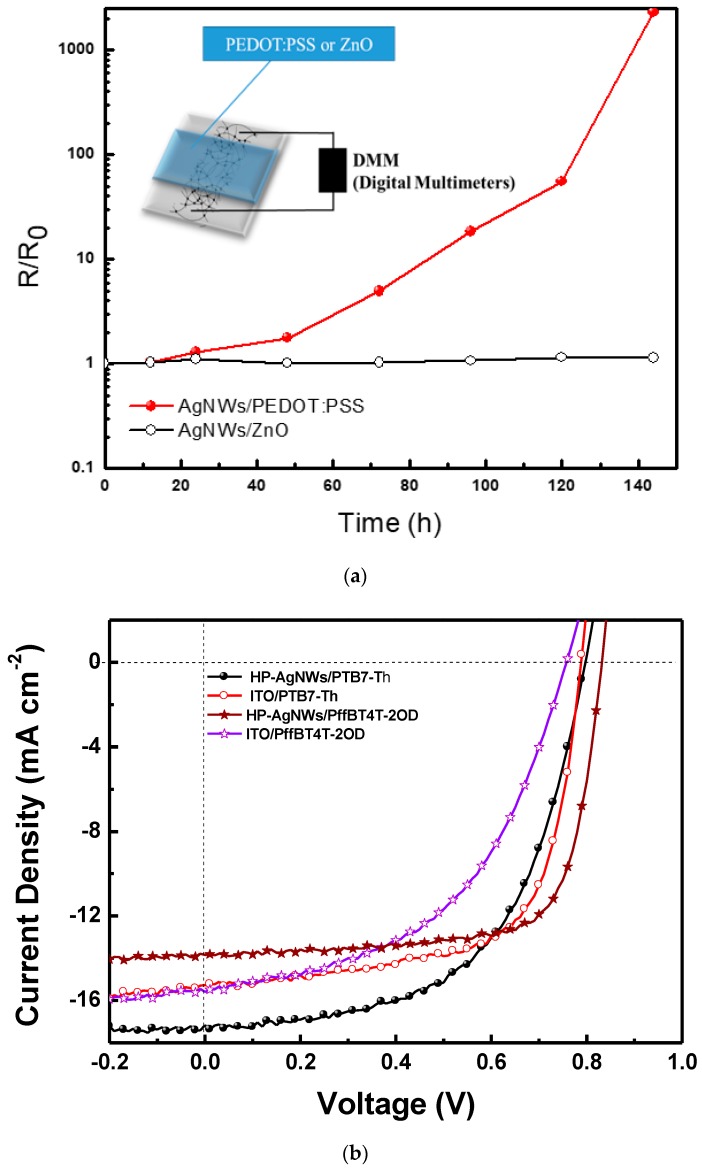
(**a**) Variation in *R*_s_ of HP-AgNW/PEDOT:PSS and HP-AgNW/ZnO films under long-term storage in air; (**b**) *J*-*V* curves of PSCs made using ITO, HP-treated AgNWs and different active layers.

Because of its acidity and its own structural inhomogeneity, PEDOT:PSS might cause the degradation mechanism and limit the life of PSCs. We evaluated the stability of the HP-AgNW/PEDOT:PSS and HP-AgNW/ZnO under room temperature/air (ca. 27 °C with an average humidity of 75%). Figure 4a shows the variation of *R*_s_ for the HP-AgNW/PEDOT:PSS and HP-AgNW/ZnO films. The inset shows the schematic of the resistance measurement for the electrodes. We found that the *R*_s_ values of the HP-AgNWs/PEDOT:PSS electrodes increased markedly. The *R*_s_ value of HP-AgNWs/PEDOT:PSS increased by almost three orders of magnitude after storing in air for 140 h, indicating its high air sensitivity. By contrast, the *R*_s_ value of the HP-AgNW/ZnO electrodes can remain constant for a long time. Because the ZnO layer is not sensitive to humidity and is a neutral material, ZnO plays the role well of a protective layer preventing AgNWs from direct exposure to air and improving stability. Figure 4b shows the *J*-*V* curves of inverted PSCs made through the active layers of PffBT4T-2OD:PC_71_BM or PTB7-Th: PC_71_BM, respectively. The *J*-*V* characteristic is listed in Table 1. The PSCs with different active layers were successfully prepared through HP treatment in this study, and their *V_oc_*, *J_sc_*, FF and PCEs values were 0.80 V, 17.17 mA cm^−2^, 0.57, 7.83% for PTB7-Th:PC_71_BM and 0.76 V, 15.59 mA/cm^−2^, 0.49, 5.81% for PffBT4T-2OD:PC_71_BM. For the efficiency of PSCs based on the ITO/glass electrode and these three active layers, PTB7-Th:PC71BM and PffBT4T-2OD:PC_71_BM were 8.03% and 7.16%, respectively. The results demonstrate that the PCEs obtained from HP-AgNW-based PSCs are close to those measured from ITO-based PSCs. We calculated the series (*R*_s_) and shunt (*R*_sh_) resistances from the respective *J*-*V* curves. The values of *R*_sh_ and *R*_s_ of the ITO/ZnO/PTB7-Th, HP-AgNWs/ZnO/PTB7-Th, ITO/ZnO/PffBT4T-2OD:PC_71_BM and HP-AgNWs/ZnO/PffBT4T-2OD devices are 540/3.3, 574/6.0, 603/2.3 and 298/7.0 Ω cm^2^, respectively (Table 1). The values of *R*_s_ are in response to their interfacial and bulk resistances [30]. The value of *R*_sh_ typically is associated with the leakage current in a device. Higher FF values are associated with greater values of *R*_sh_ and lower values of *R*_s_. The *R*_sh_ and *R*_s_ of these devices are correlated well with their FF values. 

## 4. Conclusions

Because of the trade-off between the conductivity, surface roughness and transparency of AgNW electrodes, it was challenging to fabricate a high-performance AgNW-derived flexible optoelectronics system. In this study, we used CT and HP treatment to improve the conductivity of AgNW electrodes (40–77% higher than the normal AgNW electrodes) and simultaneously maintained their high transparency (T% > 80%). The CT-and HP-derived AgNW electrodes were used to replace expensive and brittle ITO electrodes for the application in flexible PSCs. Simultaneously, the surface roughness could also be modified, which solved the problem of short circuiting for the HP-derived AgNW electrodes. We successfully demonstrated the flexible PffBT4T-20D:PC_71_BM-based PSC devices using HP-AgNW electrodes with a high performance of 5.09%, and the cell maintained more than 70% of its efficiency after a bend test (100 times). We further fabricated an AgNW/ZnO-based inverted PSC device and observed a PCE of 7.83% for the device using PTB7-Th:PC71BM as an active layer. The results obtained from this study demonstrate that the efficiency of PSCs made with AgNWs or ITO electrodes can be similar, but the cost of using AgNWs for electrodes is much lower than that using ITO. Because of the easy nature of HP treatment and the high reproducibility of the derived devices (because of lower surface roughness), the HP-AgNWs have the potential to replace ITO electrodes in the application of PSCs.

## Figures and Tables

**Table 1:** *J*-*V* characteristics of PSCs based on AgNW electrodes and three different types of active layer. PCE, power conversion efficiency.

	*V*_oc_ (V)	*J*_sc_ (mA cm^−2^)	FF	PCE (%)	*R*_s_ (Ω cm^2^)	*R*_sh_ (Ω cm^2^)
Glass/AgNWs/PEDOT:PSS/P3HT:PC_60_BM/Ca/Al	0.51	9.21	0.54	2.54	3.8	465
Glass/HP-AgNWs/PEDOT:PSS/P3HT:PC_60_BM/Ca/Al	0.54	8.98	0.61	2.96	3.5	505
Glass/ITO/PEDOT:PSS/PffBT4T-2OD:PC_71_BM/Ca/Al	0.73	14.68	0.50	5.36	3.8	325
Glass/AgNWs/PEDOT:PSS/PffBT4T-2OD:PC_71_BM/Ca/Al	0.66	15.18	0.42	4.21	13.6	179
Glass/HP-AgNWs/PEDOT:PSS/PffBT4T-2OD:PC_71_BM/Ca/Al	0.70	17.71	0.43	5.33	14.5	233
PET/HP-AgNWs/PEDOT:PSS/PffBT4T-2OD:PC_71_BM/Ca/Al	0.68	16.62	0.45	5.09	12.6	203
Glass/ITO/ZnO/PTB7-Th: PC_71_BM/MoO_3_/Ag	0.79	15.26	0.67	8.03	3.3	540
Glass/HP-AgNWs/ZnO/PTB7-Th: PC_71_BM/MoO_3_/Ag	0.80	17.17	0.57	7.83	6.0	574
Glass/ITO/ZnO/PffBT4T-2OD:PC_71_BM/MoO_3_/Ag	0.73	13.82	0.71	7.16	2.3	603
Glass/HP-AgNWs/ZnO/PffBT4T-2OD:PC_71_BM/MoO_3_/Ag	0.76	15.59	0.49	5.81	7.0	298

## Supplementary Materials

The following are available online at www.mdpi.com/2073-4360/9/11/635/s1. Figure S1: AFM topography images of (a) AgNW and (b) HP-AgNW films; Figure S2: *R*_s_ of PET/AgNWs film at different hot-pressing times and temperatures; Figure S3: UV-Vis spectra of PET/AgNWs film treated at different HP temperatures for 10 min; Figure S4: SEM images at a 45 degree inclination: (a) PET/AgNWs/PEDOT:PSS before hot-pressing treatment; (b) PET/AgNWs/PEDOT:PSS after hot-pressing treatment.
