# Supplementary material for: Comprehensive Study on Chemical and Hot Press-Treated Silver Nanowires for Efficient Polymer Solar Cell Application
Source: Polymers (Basel). 2017 Nov 22;9(11):635. doi: 10.3390/polym9110635 (PMC6418780; doi:10.3390/polym9110635)
Supplement: Supplementary file 1 [file polymers-09-00635-s001.pdf]

Supplementary Materials: Comprehensive Study on Chemical and Hot Press-Treated Silver Nanowires for Efficient Polymer Solar Cell Application

Yang-Yen Yu, Yo-Jen Ting, Chung-Lin Chung, Tzung-Wei Tsai and Chih-Ping Chen

1. Characterization and measurements

UV-Vis absorption spectra were recorded using a JASCO V-750 UV-Visible/NIR spectrophotometer. Energy levels were measured using a Riken Keiki AC2 photoelectron spectrometer (UV intensity: 50 nW). Surface morphologies of the polymer:PCBM thin films were observed through atomic force microscopy using a Nanoscope 3D Controller (MultiMode 3D SPM, Digital Instruments) operated in tapping mode at an ambient temperature; the etched Si probe exhibited a resonant frequency of 131 kHz and a spring constant of 11 $N\cdot m^{-1}$. Current–voltage (*J*–*V*) curves of PSC devices were measured using a computer-controlled Keithley 2400 source measurement unit (SMU) and a Newport solar simulator (Oriel Sol2A Class ABA Solar Simulators) under AM 1.5 G illumination. The illumination intensity was calibrated using a standard Si photodiode detector equipped with a KG-5 filter. The output photocurrent was adjusted to match the photocurrent of the Si reference cell to obtain a power density of 1000 W m^{-2}. After encapsulation, all devices were operated under an ambient atmosphere at 25 °C. The active area of each device was 10 mm^2.

2. Device fabrication

Glass/ITO substrates (Sanyo, Japan (8 Ω sq^{-1})) were patterned lithographically, cleaned and sonicated (with detergent, DI-water, acetone and isopropanol), dried on a hot plate (120 °C, 5 min) and treated with oxygen plasma for 5 min. PET substrate was used following the same cleaning and sonication process. For normal device fabrication, poly(3,4-ethylenedioxythiophene):polystyrene sulfonate (PEDOT:PSS, Baytron P-VP AI4083) was passed through a 0.45-μm filter before being deposited (thickness: ca. 30 nm) through spin-coating (3000 rpm) in air onto the ITO or AgNW substrates and dried at 110 °C for 30 min. The ZnO layer (40 nm) was prepared from a 0.5 M zinc acetate precursor solution (2-methoxyethanol). Active layer solutions of P3HT:$PC_{61}BM$ (1:0.9; 15 mg/mL), PTB7-Th:$PC_{71}BM$ (1:1.5; 10 mg/mL, with 3 vol% of 1,8-diiodooctane (DIO)) were stirred in *o*-dichlorobenzene (*o*-DCB) overnight, filtered through a polytetrafluoroethylene (PTFE; 0.2-μm) filter and then spin-coated onto the ZnO or PEDOT:PSS layers. A PffBT4T-2OD:$PC_{71}BM$ solution (donor–acceptor (D/A) ratio, 1:1.2) was formed in chlorobenzene (CB)/*o*-DCB (8:2, v/v) with 3% of DIO (PffBT4T-2OD concentration: 9 mg mL^{-1}). To ensure complete dissolution of the polymer, the mixture was stirred at 110°C (hot plate) for a minimum of 3 h. Prior to spin-coating, the PffBT4T-2OD blend solution and the substrates/PEDOT:PSS or substrates/ZnO substrate were both preheated at approximately 100°C (hot plate). In a N_2-filled glove box, the PffBT4T-2OD:$PC_{71}BM$ layers were spin-coated (800 rpm) from the warm PffBT4T-2OD blend solution onto the preheated substrates. The PffBT4T-2OD:$PC_{70}BM$ films were annealed at 90°C for 5 min and then transferred to a thermal evaporator's vacuum chamber. The active layer solution was filtered through a 0.2-μm polytetrafluoroethylene (PTFE) filter and then spin-coated on top of the ZnO and PEDOT:PSS layers. The device was completed by depositing a 30-nm-thick layer of Ca and a 100-nm-thick layer of Al or Ag at a pressure of less than 10^{-6} torr.

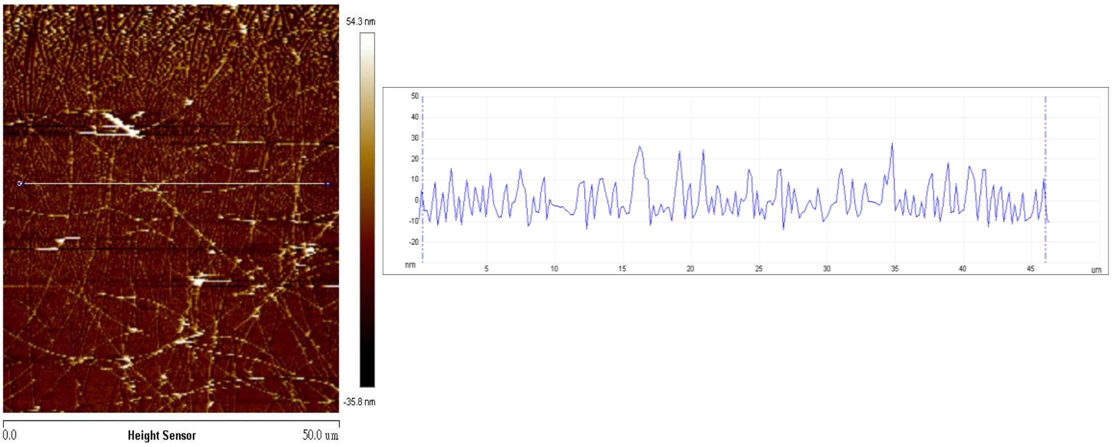

(a)

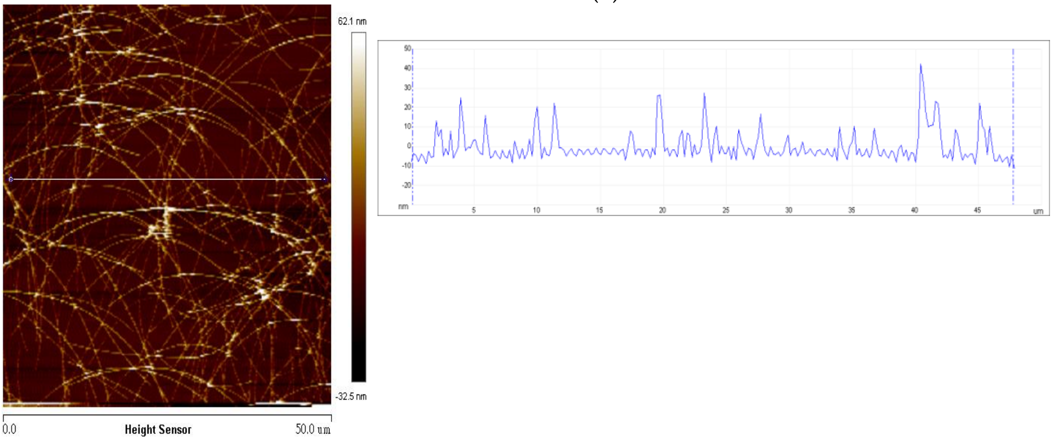

(b)

Figure S1. AFM topography images of (**a**) AgNW and (**b**) HP-AgNW films.

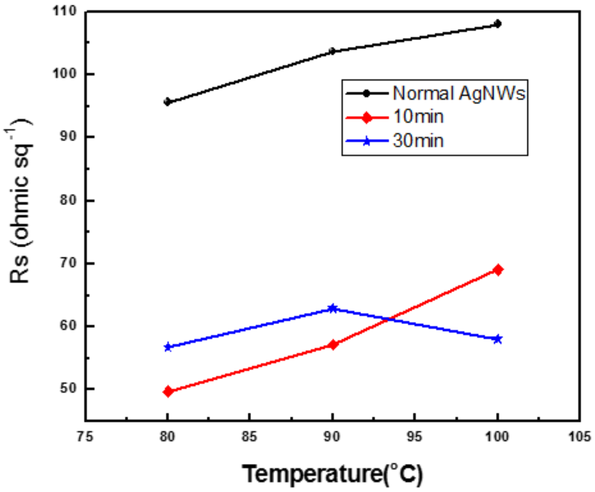

Figure S2. Rs of PET/AgNWs film at different hot pressing times and temperatures.

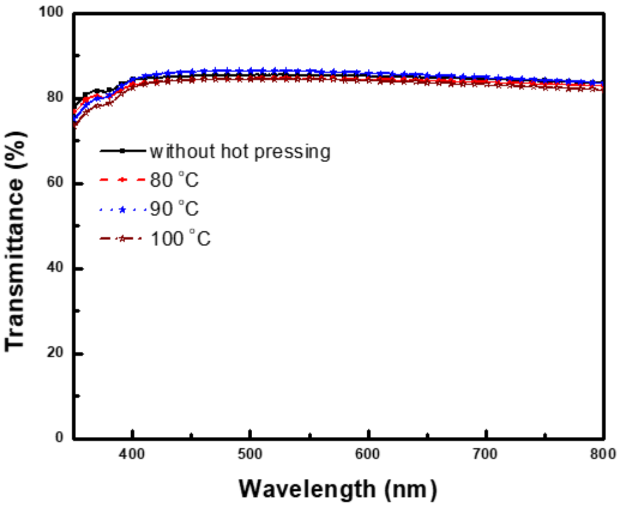

Figure S3. UV-Vis spectra of PET/AgNWs film treated at different HP temperatures for 10 min.

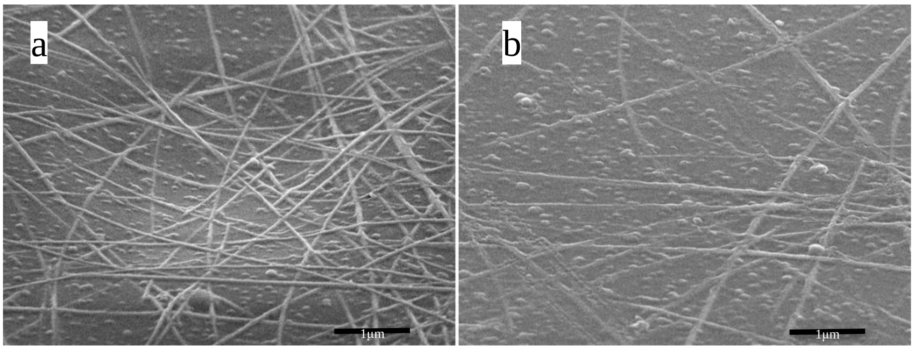

Figure S4. SEM images at a 45 degree inclination: (**a**) PET/AgNWs/PEDOT:PSS before hot-pressing treatment; (**b**) PET/AgNWs/PEDOT:PSS after hot-pressing treatment.
